# Herpes simplex virus type-1 ocular infection in thrombospondin-1 deficient C57BL/6J mice

**DOI:** 10.1371/journal.pone.0352457

**Published:** 2026-07-20

**Authors:** Aaron W. Kolb, Monica M. Sauter, Sarah Ferguson, Asha S. Jain, Nader K. Sheibani, Christine M. Sorenson, Curtis R. Brandt

**Affiliations:** 1 Department of Ophthalmology and Visual Sciences, School of Medicine and Public Health, University of Wisconsin-Madison, Wisconsin, United States of America; 2 Department of Medical Microbiology and Immunology, University of Wisconsin-Madison, Wisconsin United States of America; 3 McPherson Eye Research Institute, University of Wisconsin-Madison, Wisconsin, United States of America; 4 Department of Pediatrics, School of Medicine and Public Health, University of Wisconsin-Madison, Wisconsin, United States of America; University of Illinois at Chicago, UNITED STATES OF AMERICA

## Abstract

Herpes simplex virus type 1 (HSV-1) corneal infection triggers an immunopathological response and corneal neovascularization leading to vision impairment. Thrombospondin-1 (TSP-1) is a matricellular protein shown to regulate inflammatory processes and vascularization in the cornea. The purpose of this study was to determine if loss of TSP-1 alters the pathology of HSV-1 keratitis. The recombinant virus INV-2C or the low passage orolabial HSV-1 isolate, Ikey, were administered to scarified corneas of 8-week-old *Thbs1*^*-/-*^ and control C57BL/6J mice. Blepharitis, corneal neovascularization, corneal clouding, weight loss, viral titers, and mortality were determined on multiple days post infection. Corneal nerve loss was evaluated on day 2 and day 7. Neutralizing antibody, T-cell responses, and infiltrating cells in the aqueous and vitreous humors were quantified. There was no significant difference between *Thbs1*^*-/-*^ and C57BL/6J control mice when comparing any corneal disease outcome with either virus. Severe pan-uveitis developed in both C57BL/6J and *Thbs1*^*-/-*^ mice infected with the Ikey strain of virus. There were no significant differences in antibodies to HSV or in the number of HSV-1 specific IFN-g secreting T-cells in the spleen between C57BL/6 or TSP-1^-/-^ mice. The lack of TSP-1 did not affect the severity of corneal disease, immune responses to HSV-1, or sensory nerve loss suggesting either TSP-1 functions are not involved in HSV-1 pathogenesis or that other members of the TSP family can substitute for the loss of TSP-1.

## Introduction

Herpes simplex virus type 1 (HSV-1) is a significant human pathogen infecting between 50 and 90% of people [1–4]. HSV-1 commonly causes mucocutaneous infections at multiple sites in the body including skin, eyes, mouth, and genitals [5]. In addition, HSV-1 is the leading cause of sporadic encephalitis with a high mortality rate. For HSV-1 ocular infections, the incidence ranges from 8.4 to 31.5 per 100,000 people depending on the study [6–8] and is the leading cause of vision loss due to infectious blindness in developed countries.

Ocular HSV-1 primary infections typically present as conjunctivitis with or without corneal involvement. In some cases, corneal infection (keratitis) develops secondary to conjunctivitis. The primary infection usually clears without permanent corneal damage, particularly with the use of antivirals. However, latent infection is established in the trigeminal ganglia, and subsequent reactivation events stimulate cumulative immune responses that lead to infiltration of immune cells, corneal scarring, corneal edema, loss of sensory nerves in the cornea, and neovascularization. This results in corneal clouding and loss of vision. The loss of sensory nerves can lead to neurotropic keratitis [9–16].

Thrombospondin-1 (TSP-1) is a 450 kDa matricellular protein with multiple functions that affect angiogenesis, development and repair of corneal nerves, cell migration, wound healing, antigen presentation, and inflammation [17–19]. TSP-1 acts by binding to any of several receptors, including integrins, CD36, CD47, and glycosaminoglycans to carry out these functions [18,20] Many of these processes are potentially involved in HSV-1 corneal infection and TSP-1 is expressed in the cornea where it could influence pathogenic mechanisms and healing processes [21–25]. For example, using a suture induced corneal neovascularization model, Cursiefen et. al. [21], found that corneal angiogenesis was significantly higher in *Thbs1*^*-/-*^ mice suggesting that loss of TSP-1 would lead to increased corneal neovascularization. Under certain circumstances TSP-1 also affects antigen presentation, which could alter the immunopathology in HSV-1 keratitis (HSK) [26,27]. The TSP-1 null mice develop a form of dry eye disease beginning between 6–8 weeks of age [28]. In this model Tatematsu et. al. [19], reported that corneal sensory nerves displayed distinct structural changes so one might expect to see alterations in sensory nerve loss in HSV-1 infected *Thbs1*^*-/-*^ mice. TSP-1 is also an important activator of transforming growth factor-β (TGF-β), which is immunosuppressive and is critical for the development of Anterior Chamber Associated Immune Deviation (ACAID) [29]. Loss of TSP-1 also results in delayed corneal wound healing, thus TSP-1 loss could result in increased corneal scarring and enhanced immune cell infiltration and pathology during HSK [22].

HSV-1 infection of keratocytes, endothelial cells, smooth muscle cells, and endothelial cells down-regulates expression of TSP-1, which is consistent with the host shutoff that occurs during viral infection [30–33]. TSP-1 is also reduced in several cell types infected with human cytomegalovirus (HCMV) including retina glial cells, primary fetal astrocytes, and glioblastoma cells [22,34–37]. Kaposi’s Sarcoma associated herpesvirus (KSHV) encodes 4 microRNAs that target TSP-1 and reduce expression in HEK293 cells [38] TSP-1 is poorly expressed in Kaposi’s sarcoma lesions but it still inhibits angiogenesis, which is a critical component of the tumorigenic process [39].

Although TSP-1 has multiple functions that could affect HSK, this has not been tested *in vivo*. To determine if loss of TSP-1 altered the pathology of HSK, we infected C57BL/6J wild type and *Thbs1*^*-/-*^ mice and assessed multiple parameters of ocular infection, viral titers, and immune functions. We found that there were no significant differences in the severity of blepharitis, corneal neovascularization, stromal keratitis, viral replication, or neurovirulence. We also found no differences in the numbers of HSV-1 specific Interferon-γ (IFNγ)-secreting cells, neutralizing antibody titers, or CD4^+^ or CD8^+^ cells in the spleen. Finally, we found that the low passage clinical isolate, Ikey, induced pan-uveitis in the absence of significant keratitis in both C57BL/6J control and *Thbs1*^*-/-*^ mice. These results indicate that, at the level of clinical disease severity, TSP-1 does not play a critical role in HSV-1 keratitis. It is possible that other members of the TSP family provide redundant functions.

## Materials and methods

### Cells and virus

Vero cells (Cat# CCL-81; ATCC, Manassas, VA, USA) were grown in Dulbecco’s Modified Eagle’s Medium (DMEM) with 5% serum (1:1 Fetal Bovine Serum (GeminiBio, West Sacramento, CA, #900−108, lot A325005)/Defined Supplemented Calf Serum (HyClone, Logan, UT, #SH30072.03, lot AE29278325) at 37^0^C with 5% CO_2_. We initially used the INV-2C recombinant virus which has a 30% mortality rate in BALB/C mice [40]. To determine if a more virulent virus would reveal differences, we also tested the low passage orolabial HSV-1 isolate, Ikey, whose genome has been sequenced (GenBank OQ724957) [40]. Ikey is highly virulent in BALB/C mice causing severe blepharitis, corneal clouding, and corneal neovascularization, weight loss, and was lethal to 100% of the mice (S1 Fig). Note that mortality was not an end point and mice that were moribund were euthanized and then counted as a death. Ikey also replicated to high titers in the cornea (S1 Fig). Master viral stocks were prepared in Vero cells as we described previously and infectious titers were determined by plaque assay [41]. The titer of the INV-2C master stock was 2.0 X 10^8^ infectious units per ml. and the titer of the Ikey master stock was 1.0 X 10^9^ infectious units per ml.

BALB /C mice were infected with 1 X 10^5^ PFU of virus following corneal scarification. The severity of blepharitis, corneal clouding, corneal neovascularization, weight loss, and mortality (euthanized due to morbidity) were assessed on the days indicated (Panels A, B, and C respectively) as we have described previously [41–44]. Viral titers were assessed using plaque assays of corneal washes on Vero cells (Panel D).

### Animals

TSP-1 null (*Thbs1*^*-/-*^) mice [45] and control C57BL/6J mice (both on the C57BL6J background and lacking the Rd1 and Rd8 mutations) were bred in-house using standard procedures with water and food ad libitum.. The mice were genotyped using PCR on tail DNA and TSP-1 expression was tested using RT-PCR on RNA isolated from eyes to confirm the lack of TSP (S2 Fig). Tail DNA from one mouse each from four different litters of WT and *Thbs1*^*-/-*^ mice were amplified by PCR using TSP-1 specific primers followed by electrophoresis. All 4 WT mice had a PCR product corresponding to the WT alleles and all 4 *Thbs*^*-/-*^ had the PCR product corresponding to the mutant alleles and all mutant mice were homozygous. RT-PCR on RNA isolated from WT and *Thbs*^*-/-*^ revealed significant expression in the WT mice but no expression in the *Thbs*^*-/-*^ mice confirming the KO phenotype (S2 Fig). To quantify corneal disease, weight loss, mortality, and viral replication there were 15 mice per group of both sexes for the INV-2C virus. For the same studies using the Ikey strain we used 14 C57BL/6J control and 19 *Thbs1*^*-/-*^ mice of both sexes. Both sexes were used but the numbers of each sex were not enough to statistically compare sex differences. Previously published work has shown that sex differences are not involved in the severity of HSV-1 ocular infection in C57BL/6 or BALB/C mice [46]. The numbers of mice used for each analysis are indicated in the Figure legends. Sustained release Buprenorphine was administered at the time of infection and continued for 7 days for pain relief. Euthanasia was carried out by anesthetizing the mice with 3–5% isoflurane followed by cervical dislocation, which was approved by the UW-Madison IACUC (Protocol Number: M006440) and conformed to the ARVO guidelines for the use of animals in research and the Guide for the Care and use of Laboratory Animals of the National Institutes of Health. The *Thbs1*^*-/-*^ mice develop a dry eye syndrome resembling Sjogren’s Syndrome beginning between 6–8 weeks of age so all studies were done with 8-week-old mice to avoid potential complications from the autoimmune disease [17, 47].

Transgene screening and expression. (A) The identity of wild type and *Thbs1*^*−/−*^ mice were confirmed by PCR screening of DNA prepared from tail biopsies using the following primers: KONEOS: 5’-TGC TGTCCATCTGCACGAGACTAG; KOTSPAS:5’-GAGTTTGCTTGTGGTGAACGCTCAG-3’; and KOTSPS: 5’-AGGGCTATGTGGAATTAATATCGG-3’. On the right, DNA from confirmed *Thbs1 + /−* and C57BL6/j (+/+) mice were run for comparison. (B) The expression of transgene (*Thbs1*) was confirmed by qPCR analysis of cDNAs prepared from total RNA extracted from the mouse retina using the following primers: Thbs1F: 5’-TGGCCAGCGTTGCCA-3’; Thbs1R: 5’-TCTGCAGCACCCCCTGAA-3.’ The house keeping gene Rpl13a was used as control: Rpl13aF: 5’-TCTCAAGGTTGTTCGGCTGAA-3’ and Rpl13aR: 5’-GCCAGACGCCCCAGGTA-3.’ Samples were prepared from one mouse from four different litters. The details of PCR and qPCR analysis were as previously described [48–50].

### Infection and ocular disease scoring

Ocular infection and disease scoring was described previously [41–44]. Briefly, the right corneas of 8-week-old mice were scarified with a 30 ga. needle, followed by application of a 5 µl drop of DMEM with 2% serum (1:1 fetal bovine serum/defined supplemented calf serum) containing 1 X 10^6^ PFU of virus. The infected eyes were examined on the days post-infection (PI), as noted in the figures, using a Wild-Heerbrugg (Heerbrugg, SG, Switzerland) surgical microscope. The severity of blepharitis, corneal neovascularization, and corneal clouding (stromal keratitis) was scored as follows. Blepharitis: 1 + , mild swelling of eyelids; 2 + moderate swelling with some crusting; 3 + eyelid swollen 50% shut with severe crusting; 4 + eye swollen and crusted shut. Corneal neovascularization: 1 + , less than 25% corneal involvement (vessel ingrowth from the limbus); 2 + , 25–50% corneal involvement; 3 + , > 50% corneal involvement. Corneal clouding: 1 + , some haze but iris detail visible; 2 + , moderate haze, iris detail obscured; 3 + , cornea totally opaque; 4 + cornea perforated. A score of 0 indicated no observable pathology. Weights were recorded at baseline and on each scoring day. and mortality was determined by counting mice that were moribund and had to be sacrificed.

### Measurement of ocular viral titers

Tear film samples were collected by flushing the cornea with 10µl of DMEM with 2% serum (1:1 defined supplemented calf serum/fetal bovine serum), penicillin, streptomycin, and amphotericin B, and adding the wash to 190 µl of the same medium. Samples were stored at −80^0^C. Viral titers were quantified by serial 10-fold dilution and plaquing on Vero cells.

### Corneal nerve loss

The eyes from C57BL/6J and *Thbs1*^*-/-*^ mice were harvested on days 2 and 7 PI and fixed in 4% paraformaldehyde. The pigment was removed by submerging the eyes in 15% hydrogen peroxide at 55°C for 2 hours. The eyes were then washed with PBS 3 times for 5 minutes. Following washing, the eyes were incubated in X-CLARITY hydrogel:initiator solution (cat# C1310X; Logos Biosystems, Annandale, VA, USA) overnight at 4°C. They were then polymerized at 37°C in a −70 kPa vacuum for 3 hours using the X-CLARITY Polymerization System (cat# C20001; Logos Biosystems). The ocular samples were washed 3 times for 5 minutes with PBS buffer, then cleared using the X-CLARITY Tissue Clearing System II (cat# C30001; Logos Biosystems) with a pump speed of 50 rpm, and 1 Amp current at 37°C for 20 hours. The eyes were then washed 3 times for 5 minutes in PBS.

Following tissue clearing, the intact eyes were subjected to immunofluorescence staining. The samples were blocked with PBS + 10% FBS for 24 hours at 37°C, then incubated with a 1:300 dilution of β-III-tubulin antibody (cat# PA5–85639; ThermoFisher, Waltham, MA) in blocking buffer for 72 hours at 37°C. The eyes were then washed 3 times for 2 hours with PBS and incubated for 72 hours at 37°C with a 1:300 dilution goat anti-rabbit Alexa Fluor 594 (cat# A-11012; ThermoFisher, Waltham, MA) in blocking buffer. The eyes were subsequently washed 3 times for 2 hours in PBS, then imaged with an Andor XD confocal spinning disk microscope (Andor, Belfast, NI, UK). After imaging, the number of β-III-tubulin expressing corneal nerve segments were counted in 5 random fields from 3 mice.

### FACS analysis

Following sacrifice at 28 days PI, mouse spleens were harvested and placed into petri dishes containing HBSS (Hank’s balanced salt solution) on ice prior to processing. The spleens were then minced, and the cells were passed through 40µm cell strainers (#352350, Falcon, Corning, NY) to obtain single cell suspensions. The cells were washed in PBS, then centrifuged at 600 xg for 5 minutes at 4°C. They were then resuspended in ice cold ACK lysis buffer (0.15M ammonium chloride, 0.01M potassium bicarbonate, 0.0001M EDTA), centrifuged 5 minutes at 600g, then washed with cold phosphate buffered saline (PBS). Following the PBS wash, the spleen cells were centrifuged at 600 xg for 5 minutes, then split into two portions, one for ELISPOT assay and the other for FACS analysis. For the ELISPOT assay, 1x10^5^ cells were plated in triplicate with three replicates for each sample, while 1x10^6^ cells were used in triplicate per sample for FACS analysis.

For FACS analysis the spleen cells were resuspended in PBS + 0.5% bovine serum albumin (BSA), then paraformaldehyde was added (1% final concentration) and incubated at room temperature for 15 minutes. The cells were centrifuged at 600 xg for 5 minutes, then resuspended in cold PBS + 20% bovine calf serum. Fc receptors were blocked with 1 µL per 1x10^6^ spleen cells of anti-mouse CD16/32 antibody (BioLegend, San Diego, CA; Cat#101319) for 20 minutes at 4°C. The cells were then stained with either anti-CD4 (BioLegend; Cat# 100509) or anti-CD8a FITC conjugated antibody (1 µL per 1x10^6^ cells; BioLegend; Cat# 100705) or IgG2a isotype control (BioLegend; Cat# 400506) for 20 minutes at 4°C. The samples were centrifuged at 300 xg for 5 minutes at 4°, then washed in PBS + 0.5% BSA, re-centrifuged, then resuspended in PBS + 0.5% BSA. An Attune NxT cytometer (ThermoFisher, Waltham, MA) was used to analyze the stained cells and Floreada.io (https://floreada.io; accessed April 2025) was used to visualize the results. Note, the critical comparison is between the infected C57BL/6J mice and infected TSP-1^-/-^ mice and not non-infected vs. infected mice so to reduce the number of animals we did not include uninfected mice.

### Neutralizing antibody

Blood was collected from anesthetized mice via cardiac puncture 28 days PI. Three samples were taken from C57BL/6J control mice and three samples from *Thbs1*^*-/-*^ mice. The whole blood samples were centrifuged at 2000 xg for 10 minutes at 4°C, then the serum phase was collected and stored at −80°C. For the plaque reduction assay, the serum samples were thawed, then diluted 1:2, 1:4, 1:8, 1:16, 1:32, 1:64 and 1:128. The serum dilutions were then mixed with 100 plaque forming units (PFU) of HSV-1 Ikey and incubated at 37°C for one hour. Following incubation, the serum and virus preparations were plated onto Vero cells (CCL-81; ATCC, Manassas, VA, USA) in 6-well plates, incubated to allow for plaque formation, stained with crystal violet, and counted. Each mouse was analyzed separately.

### HSV-1 specific T Cells

For the ELISPOT assay, 1 X 10^5^ cells from each spleen were seeded into triplicate wells of a mouse IFN-γ assay plate (R&D Systems, Minneapolis, MN; Cat# EL485). For secondary stimulation, 12 µg of heat-inactivated gradient purified HSV-1 viral preparation was added to each well and the plate was incubated for three days to allow IFN-γ secretion to occur. The wells were washed, secondary antibody was added, and the plate was incubated at 4°C overnight. After washing, the substrate was added according to the manufacturer’s instructions, and the number of IFN-g positive cells were counted.

### Quantification of infiltrating cells

Strain Ikey infected eyes were harvested on day 7 post-infection, fixed in 4% paraformaldehyde, paraffin embedded and sectioned. Sections were stained with hematoxylin-eosin and photographs were obtained at a magnification of 40X using a Zeiss Axiocam 702 mono camera on a Zeiss Axio Imager.Z2 microscope with Zeiss ZEN 2.3 pro software (Zeiss Microimaging, Oberkochen, Germany). The photographs were then tiled and the total number of infiltrating cells in the aqueous and vitreous chambers were counted. The eyes from 3 wild type and 3 mutant mice were counted.

### Statistical analysis

Ocular disease scores were analyzed using the Mann-Whitney U test. Sigma plot 11 (Graffiti LLC, Palo Alto, CA) was used to perform ANOVA statistical analysis on β-III-tubulin positive corneal nerve counts. Two tailed t-tests (Excel, Microsoft, Redmond, WA) were used to evaluate the neutralizing antibody titers, ELISPOT data, and infiltrating cell count experiments. For all analyses, p ≤ to 0.05 was considered significant.

## Results

TSP-1 deficient mice have defects in activation of TGF-β which results in loss of the ocular immune privilege phenomenon known as ACAID, through loss of T_regs_ [27, 51]. The loss of suppression results in inflammatory infiltration of the lacrimal glands causing a dry eye syndrome substitute for Sjogren’s syndrome that begins between 6−8 weeks of age [27]. Therefore, to avoid potential confusion between HSV-1 infection and the dry eye syndrome, we used 8-week-old mice for these studies.

### Ocular disease severity

**Fig 1:**
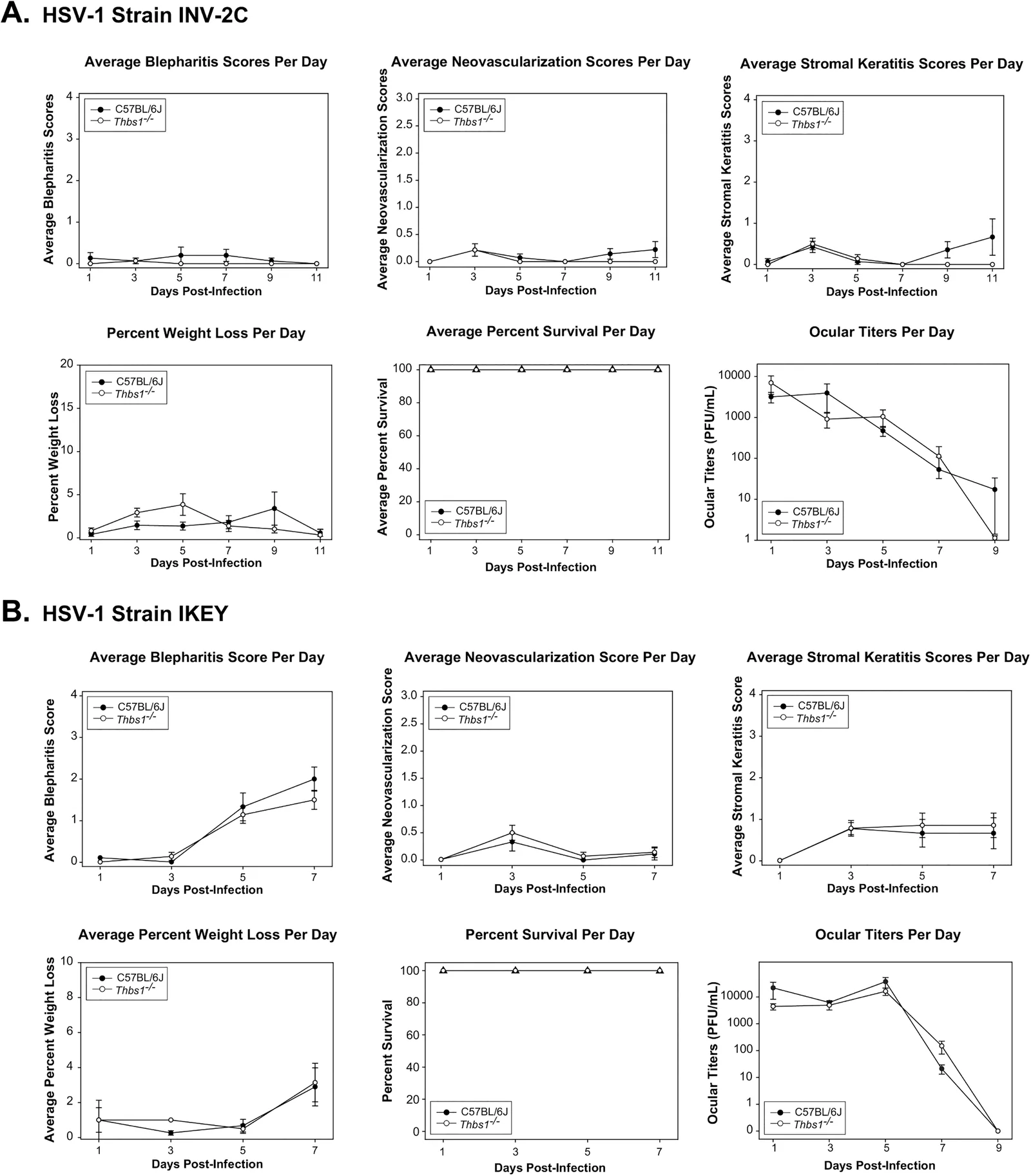
Characterization of keratitis, disease severity and viral replication in C57BL/6J and *Thbs1*^*-/-*^ mice. Two viral strains, INV-2C and Ikey, with different virulence profiles were used. For the INV-2 study there were 15 mice of both sexes in each group. For the Ikey strain we used 14 C57BL/6J and 19 *Thbs1*^*-/-*^ mice of both sexes. Mice were infected with 1 X 10^6^ PFU of virus following corneal scarification. Multiple disease characteristics were quantified on the days indicated. (A) INV-2C virus. (B) Ikey virus. Note that the scoring of eye disease was halted at day 7 PI for Ikey infected eyes because they were sacrificed for histology and immune studies. Average scores or values on each day were compared using the Mann-Whitney test.

We initially infected mice with the recombinant HSV-1 virus INV-2C which has a mortality rate of 30% in BALB/C mice [41]. As shown in Fig 1A, there was very little blepharitis, corneal neovascularization, corneal clouding, weight loss, or mortality at any time post-infection. Viral titers indicated that the INV-2C virus replicated in the cornea but there were no significant differences between the wild-type C57BL/6J control or *Thbs1*^*-/-*^ mice for any of the outcomes that were tested.

To determine if differences could be discerned with a more virulent virus, we repeated the infections with the Ikey strain of HSV-1 [41]. Ikey is a low passage oral isolate that has a mortality rate of 100% in BALB/C mice (S1 Fig). Following infection, we scored the severity of multiple disease parameters. Scoring was not continued past day 7 because the mice were sacrificed for the histology and immunology studies that are described below. As shown in Fig 1B, there was very little blepharitis, corneal neovascularization, corneal clouding, weight loss, and mortality. Viral titers indicated that the Ikey virus replicated in the cornea but there were no significant differences for any of the parameters that were measured

### Sensory nerve loss in the cornea

**Fig 2:**
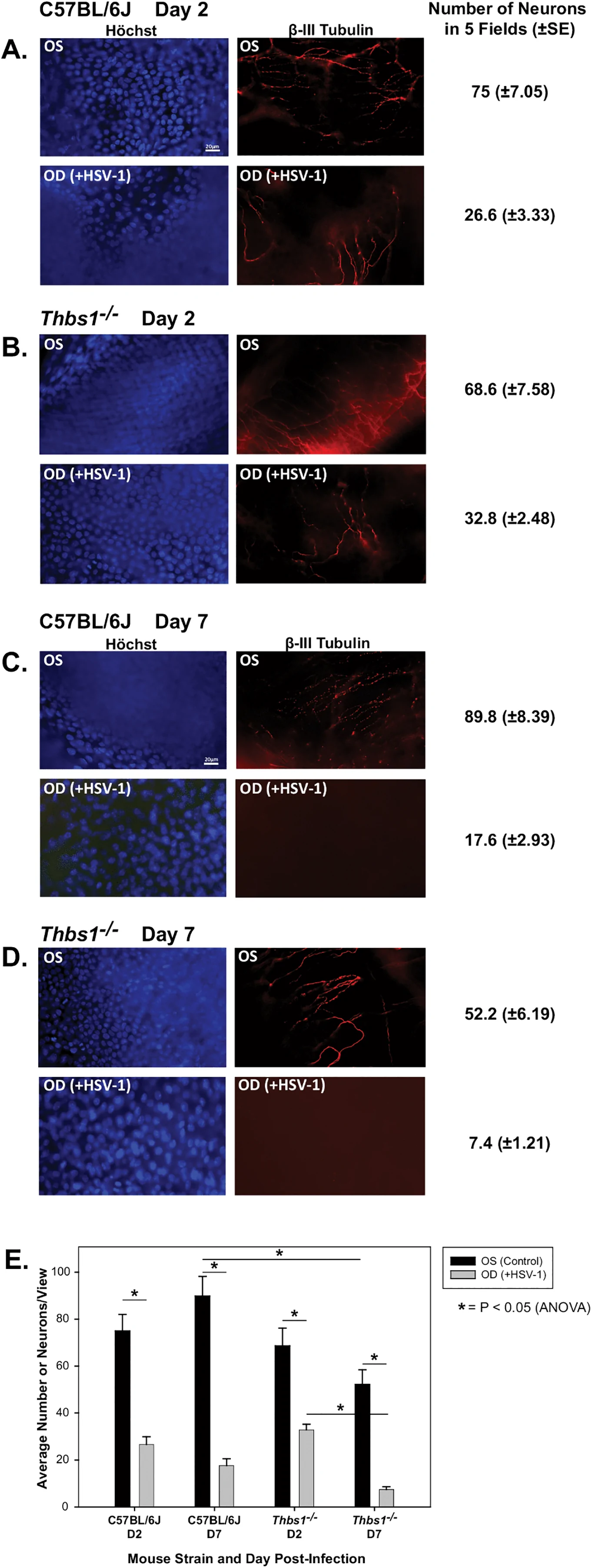
Quantification of corneal nerve loss. Whole globes from Ikey infected mice were collected on day 2 and day 7 PI, clarified using the X-Clarity system, and stained with antibody to β-III tubulin to mark axons. The globes were then imaged using a spinning disk confocal microscope. The number of nerve segments in 5 fields each from 3 different eyes were then quantified and averaged. Statistical significance was determined using ANOVA with p < 0.05 being significant. Panels A through D show representative examples of the corneal nerves and Panel E shows the statistical comparisons with p = 0.05 being significant.

Loss of TSP-1 has been shown to result in alterations in nerves in the dry eye model [19] and HSV-1 is known to induce sensory nerve loss in infected corneas [52] To assess if the lack of TSP-1 altered corneal sensory nerves in infected corneas, we harvested eyes from day 2 and day 7 Ikey infected mice, performed clarification using the X-Clarity system, stained with antibody to βIII-tubulin, and quantified the number of nerve fiber segments. Representative examples of nerve loss and quantification of nerve fiber segments are shown in Fig 2. On day 2 PI, in uninfected corneas there were 75±7.05 and 68.6±7.58 fiber segments in the C57BL/6J control and *Thbs1*^*-/-*^ mice respectively. In the infected corneas on day 2 PI, there were 26±3.33 and 32.8±2.48 fiber segments in the C57BL/6J and *Thbs1*^*-/-*^ mice respectively indicating that substantial nerve loss occurs by day 2 PI in both types of mice. Nerve loss on day 2 PI was significantly different between infected and uninfected mice but was not significantly different between the infected C57BL/6J control and *Thbs1*^*-/-*^ mice. Similar results were found on day 7, however, there was a statistically significant greater nerve loss in the contralateral uninfected *Thbs1*^*-/-*^ eyes compared to the C57BL/6J uninfected contralateral eyes. There was also a significant reduction in corneal nerves in infected day 7 vs. day 2 *Thbs1*^*-/-*^ eyes for both types of mice.

### HSV-1 immune responses

**Fig 3:**
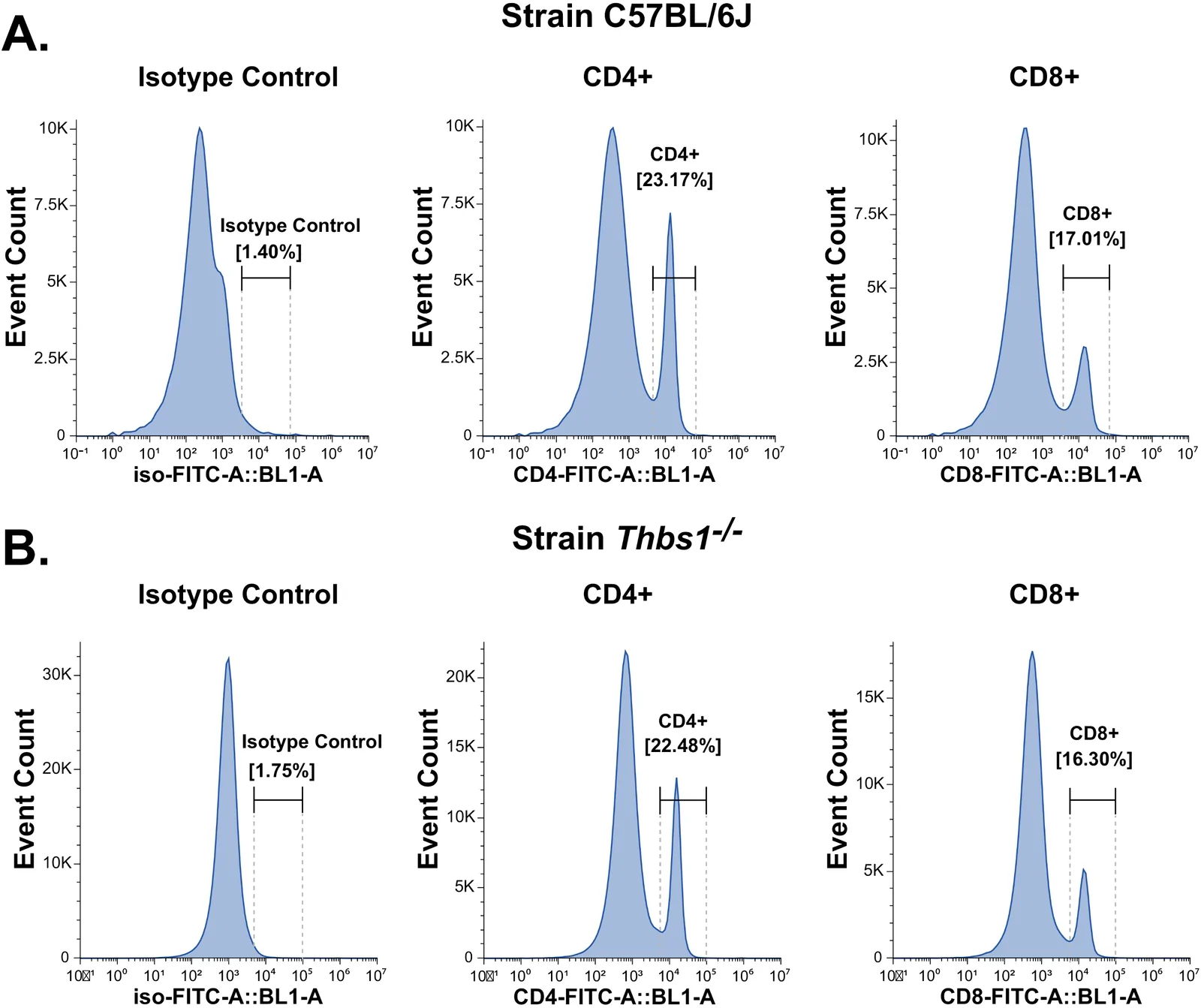
Quantification of splenic CD4^+^ and CD8^+^ cells at one month PI in mice infected with the Ikey strain. Spleens were harvested at one month PI and single cell suspensions were prepared. The cells were fixed and then stained with antibodies specific for CD4^+^ and CD8^+^ and quantified by flow cytometry as described in Materials and Methods. A non-specific isotype control was also included. N = 3.

Given that loss of TSP-1 reduces T_reg_ responses, we expected that loss of suppression should lead to increased immunopathology. We therefore measured neutralizing antibody titers, the total number of CD8^+^ and CD4^+^cells in the spleen, and the number of splenic HSV-1 specific IFN- γ-secreting T-cells. FACS analysis revealed that CD4^+^and CD8^+^T-cells constituted 23.17% and 17.01% of the cells in the C57BL/6J control mice respectively (Fig 3A). The *Thbs1*^*-/-*^ mice had 22.48% CD4^+^cells and 16.30% CD^+^ cells and these numbers were not significantly different between the C57BL/6J control and *Thbs1*^*-/-*^ mice (Fig 3B).

**Fig 4:**
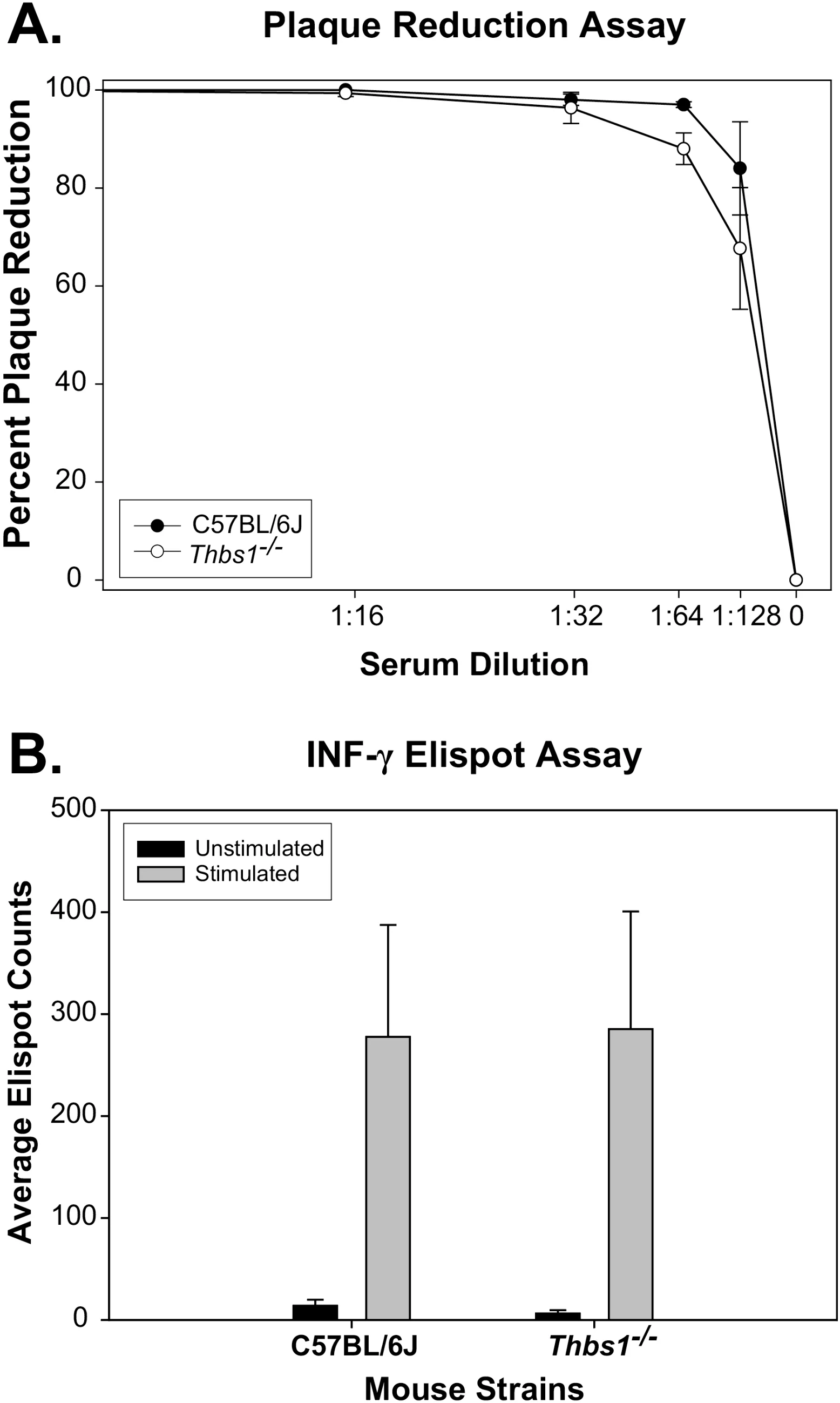
Quantification of HSV-1 specific immune responses. (A) At 28 days PI, serum was obtained from the Ikey infected mice via cardiac puncture. The serum samples were serially diluted 1:2 and mixed with 100 PFU of the Ikey strain. A plaque reduction assay was then conducted and the percentage reduction in the number of plaques was averaged. (B) To quantify HSV-1 specific IFN-g secreting cells, single cell spleen suspensions were prepared, and a commercially available IFN-g ELISPOT kit was used to quantify the number of positive cells. The data are reported as the average number of positive spots per 1 X 10^5^ splenocytes and were analyzed using a two tailed t-test with p = 0.05 being significant. N = 3.

As shown in Fig 4A, the 50% antibody neutralization titers for both the C57BL/6J control and *Thbs1*^*-/-*^ mice were greater than 1:128 and were not significantly different. The IFN-g ELISPOT results are shown in Fig 4B. In the absence of secondary antigen stimulation, there were very few IFN-γ positive cells and the differences between the C57BL/6J control and *Thbs1*^*-/-*^ mice were not significant. In the presence of HSV-1 antigen stimulation, there were approximately 300 IFN-γ positive cells per 1 X 10^5^ splenocytes in both the C57BL/6J control and *Thbs1*^*-/-*^ mice and the results were not significantly different. When considered together, the lack of TSP-1 has little, if any, effect on the immune responses to HSV-1 infection, at least at the level of analysis that was performed.

### Uveitis

**Fig 5:**
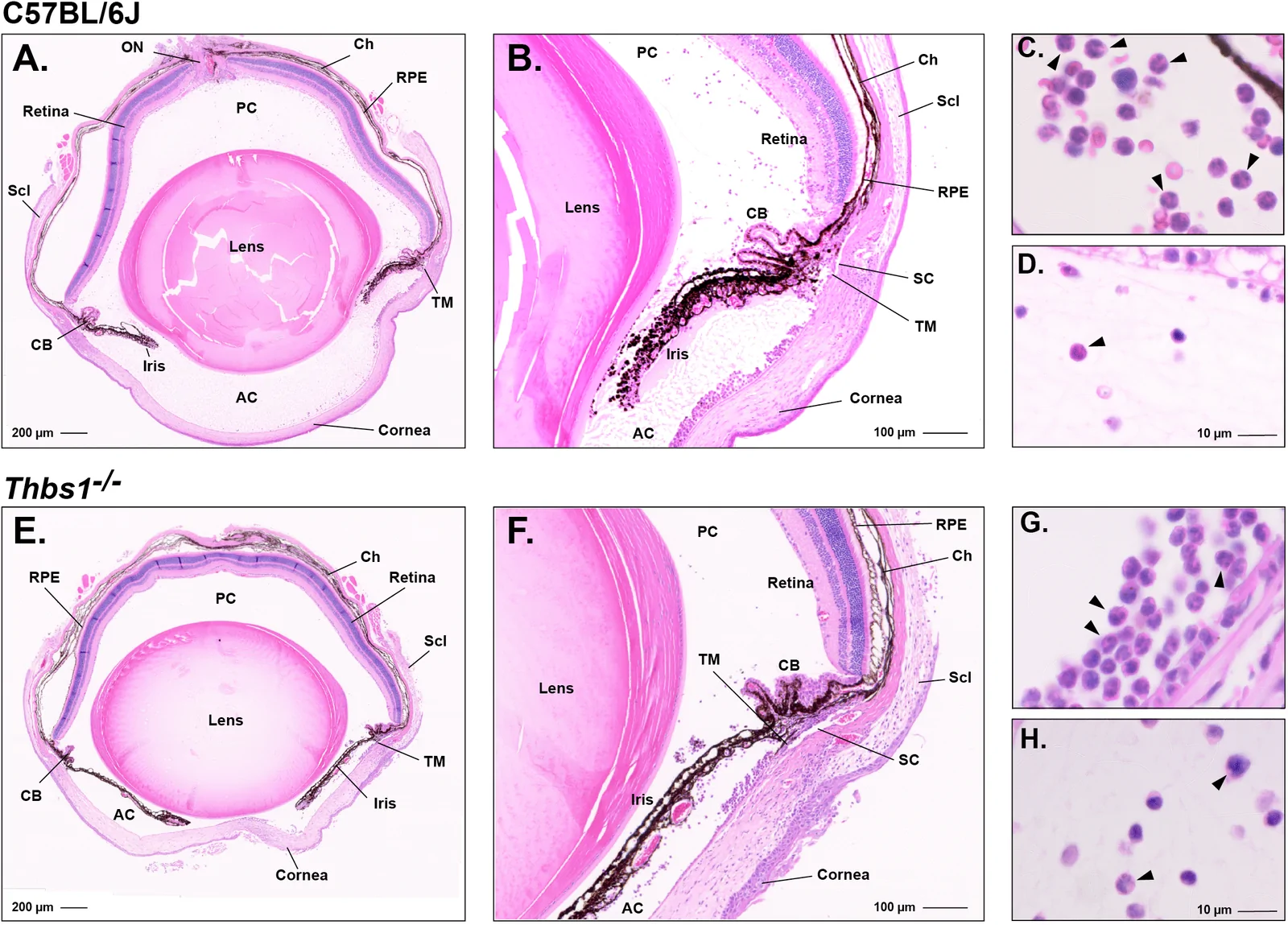
Anterior and posterior uveitis in Ikey infected mice. Eyes were harvested at day 7 PI, fixed in 4% paraformaldehyde, paraffin embedded, sectioned and stained with H & E. The H & E images were tiled, and the total number of infiltrating cells were counted for each of 3 mice. (A) Lower magnification view of a representative C57BL/6J infected eye. Panel B, higher magnification view of the angle including retina and cornea in a C57BL/6 mouse. Panels C aqueous and D vitreous show higher magnification views with arrows denoting neutrophils. Panel E) Low magnification view of a representative *Thbs1*^*-/-*^ eye. Panel F, higher magnification view of the angle with the retina and cornea in a *Thbs1*^*-/-*^ mouse eye. Panels G and H, higher magnification views of the infiltrate in the anterior segment (G) and the vitreous (H) with arrows denoting neutrophils. AC, anterior chamber; CB, ciliary body; Ch, choroid; ON, optic nerve; PC, posterior chamber; RPE, retinal pigment epithelium; Scl, sclera; TM, trabecular meshwork.

Histological analysis revealed that immune cell infiltration was present in the aqueous and vitreous of infected eyes indicating that the Ikey strain of HSV-1 caused pan-uveitis. We used H&E-stained sections to count the number of infiltrating cells in the eyes of 3 wild type and 3 Thbs^-/-^ mice on day 7 PI. Representative examples of the stained sections from C57BL/6J control mice and *Thbs1*^*-/-*^ mice are shown in Fig 5. Quantification of the number of infiltrating cells in the anterior chamber revealed there were 260 ± 205 cells in the C57BL/6 mice and 200 ± 146 cells in the Thbs^-/-^ mice (p = 0.485). In the posterior chamber there were 254 ± 251 cells in the C57BL/6 mice and 200 ± 253 cells in the Thbs^-/-^ mice (p = 0.65). Thus, there were no significant differences between the two strains of mice most likely because of the highly variable cell counts. Based on the H&E staining and morphology, the infiltrate in both types of mice was predominantly composed of neutrophils (Fig 5, arrow heads) which is consistent with previous reports on the kinetics of polymorphonuclear neutrophil (PMN) infiltration into HSV-1 infected corneas at day 7 PI. [11,13,14]

## Discussion

The pathological responses involved in HSV-1 ocular infection involve multiple factors including the genetic makeup of the virus, innate immune responses at early phases of the infection, mechanisms involved in antigen presentation, alterations in the control of corneal vascularization, the generation of adaptive immune responses, migration of inflammatory cells into the infected cornea, and altered wound healing responses leading to corneal scarring [14,15,43 13,16,53,54].

The Thrombospondin protein family (TSPs-1–5) are large matricellular proteins that play important roles in multiple cellular processes that could affect the pathogenesis of HSV-1 ocular infection. TSP-1 and 2 are closely related and constitute the subgroup A TSP’s [55 17,18,20]. TSP-1 is a major activator of TGF-β1, which plays an important role in immune suppressive functions in the eye by promoting the generation of T_reg_ cells (ACAID) and regulating the development of macrophages, dendritic cells, and granulocytes [29,56,57]. Thus, a lack of TSP-1 could result in enhanced inflammation. TSP-1 also inhibits vascularization by limiting release of VEGF from the extracellular matrix and directly binding VEGF, so enhanced corneal neovascularization could occur in the absence of TSP-1 [58–61]. Additional corneal scarring could be expected in TSP-1 null mice given this protein’s involvement in TGF-β regulation and previously described alterations in would healing [62,63]. Finally, TSP-1 plays a role in development and repair of corneal nerves so HSV-1 induced loss of sensory nerves in the cornea could be more severe in its absence [19].

Given the known pathological processes involved in HSV-1 ocular infection and the known functions of TSP-1, we hypothesized that the pathogenesis of HSV-1 corneal infection would be enhanced in *Thbs1*^*-/-*^ mice. Surprisingly, we found the severity of corneal disease was not significantly different between wild-type and *Thbs1-/-* mice. We also found no significant differences in viral replication, weight loss or neurovirulence. Corneal nerve loss was also not significantly different between infected C57BL/6J control and *Thbs1*^*-/-*^ mice, however, we found a significant difference in corneal nerves on day 7 in the uninfected contralateral C57BL/6J control and *Thbs1*^*-/-*^ eyes which is consistent with previous findings [19]. Titers of HSV-1 neutralizing antibody were essentially identical between C57BL/6J control and *Thbs1*^*-/-*^ mice at the dilutions that were tested. Analysis of CD4^+^ and CD8^+^ cell numbers in the spleen were not significantly different and there were no significant differences in the number of HSV-1 antigen specific IFN-g secreting cells in the spleen. Thus, despite the described roles for TSP-1 in regulating immune functions, its absence does not appear to make a difference in HSV-1 keratitis and the antiviral immune responses that were measured.

One potential explanation for the lack of difference we saw is that one or more of the other TSP’s is at least partially substituting for the loss of TSP-1. TSP-1 is part of a family of matricellular proteins (TSPs-1–5) and is divided into two subgroups, A and B, based on structure [55]. TSP-1 and TSP-2 belong to subgroup A and are related structurally, although they have different functions. Given the structural similarity, it is possible that TSP-1 and TSP-2 have some redundant functions. TSP-2 is less well studied so whether it has redundant functions with TSP-1 relevant for HSV-1 keratitis is not known and will require additional studies. TSP-2 KO, and TSP-1/2 double KO mice are available so studies using these mice might be informative regarding potential overlapping functions. Another possibility is that TSP-1 functions are not critical in HSV-1 ocular infections.

TSP-1 is expressed in the cornea and is found in the basal epithelium, Decemet’s membrane, and endothelium where it plays a critical role in maintaining corneal angiogenic and lymphangiogenic privilege by inhibiting development of lymphatics and blood vessels [17, 64]. The inhibition of new vessel formation involves TSP-1 binding to CD36 on pro-lymphangiogenic macrophages, which inhibits VEGF-C and -D expression [17,28]. *Thbs1*^*-/-*^ mice do not develop spontaneous lymphangiogenesis, consistent with redundant mechanisms maintaining corneal clarity but inflammatory conditions can override this, so it is surprising that we didn’t see differences in neovascularization. In our experience, corneal neovascularization in C57BL/6 mice occurs following HSV-1 infection but the vessels infiltrate only a short distance (1–2 mm) compared to BALB/C mice where the entire cornea can be involved (unpublished observations). Corneal neovascularization also depends on the viral strain [45]. Thus, C57BL/6J mice appear to be innately more resistant to neovascularization, and this could explain why we did not see significant differences. The redundant functions maintaining corneal clarity might also explain why we saw no differences in neovascularization.

TSP-1 plays a critical role in regulating immune responses, including effects on antigen presentation and cell migration [17], so we expected to see differences in immunopathologic diseases like HSK. Antigen presenting cells (APCs) in the anterior chamber are exposed to high concentrations of TGF-β which alters their maturation [65,66] TGF-β increases TSP-1 expression on ocular APCs resulting in the inhibition of CCR7 expression [67] which subsequently inhibits migration to draining lymph nodes and potentially shifting migration to the spleen [68]. In the spleen, TSP-1 binding to CD47 can enhance interactions between APCs and T-cells and localized secretion of TGF-β in this context plays a role in promoting the generation of T_reg_ cells [17,68]. We expected that the loss of TSP-1 would prevent this regulatory response resulting in enhanced corneal inflammation, but this was not the case. The systemic immune suppression is typically generated following anterior chamber injection of antigen, so corneal infection bypasses the suppressive response [56].

The absence of TSP-1 results in dysregulation of the immune system. *Thbs1*^*-/-*^ mice spontaneously develop autoreactive T-cells that infiltrate the lacrimal gland like Sjogren’s syndrome (a form of dry eye disease) starting between 6 and 8 weeks of age. The T-cell infiltration in the lacrimal gland is predominantly Th17-mediated but elevated IFN-g is also present suggesting both Th17 and Th1 mechanisms are involved [69]. In animal models of experimental autoimmune uveitis (EAU), using immunization with an ocular antigen, the uveitis results from a complicated interaction involving IL-23, dendritic cells, γδ T cells, IL-1β, IFN-γ, and IL-17 and can be driven by either Th17 or Th1 responses [70–75]. Thus, it was surprising that we didn’t see enhanced inflammation in the *Thbs1*^-/-^ mice.

### Concluding remarks

Although TSP-1 regulates several processes that could be involved in the pathogenesis of HSV-1 keratitis, we found that there were no significant differences between keratitis severity, nerve fiber loss, or in antibody or T-cell responses in infected mice. This suggests that TSP-1 is not involved in these processes during this infection. Alternatively, TSP-1 is a member of a family of proteins, and we can’t rule out that redundant functions of other family members can substitute for the loss of one family member.

## Supporting information

S1 Fig Virulence characterization of strain Ikey in BALB/C mice. BALB/C mice were infected with 1 X 105 PFU of virus following corneal scarification. The severity of blepharitis, corneal clouding, corneal neovascularization, weight loss, and mortality (euthanized due to morbidity) were assessed on the days indicated (Panels A, B, and C respectively) as we have described previously. 41–44 Viral titers were assessed using plaque assays of corneal washes on Vero cells (Panel D).
(TIF)

S2 Fig Genotyping and TSP-1 expression analysis of the Thbs1-/- mice. Transgene screening and expression. (A) The identity of wild type and Thbs1 − / − mice were confirmed by PCR screening of DNA prepared from tail biopsies using the following primers: KONEOS: 5’-TGC TGTCCATCTGCACGAGACTAG; KOTSPAS:5’-GAGTTTGCTTGTGGTGAACGCTCAG-3’; and KOTSPS: 5’-AGGGCTATGTGGAATTAATATCGG-3’. On the right, DNA from confirmed Thbs1 + /− and C57BL6/j (+/+) mice were run for comparison. (B) The expression of transgene (Thbs1) was confirmed by qPCR analysis of cDNAs prepared from total RNA extracted from the mouse retina using the following primers: Thbs1F: 5’-TGGCCAGCGTTGCCA-3’; Thbs1R: 5’-TCTGCAGCACCCCCTGAA-3.’ The house keeping gene Rpl13a was used as control: Rpl13aF: 5’-TCTCAAGGTTGTTCGGCTGAA-3’ and Rpl13aR: 5’-GCCAGACGCCCCAGGTA-3.’ Samples were prepared from one mouse from four different litters. The details of PCR and qPCR analysis were as previously described.48–50.
(TIF)
