# Correction: Herpes simplex virus type-1 ocular infection in thrombospondin-1 deficient C57BL/6J mice

**DOI:** 10.1371/journal.pone.0356738

**Published:** 2026-08-20

**Authors:** Aaron W. Kolb, Monica M. Sauter, Sarah Ferguson, Asha S. Jain, Nader Sheibani, Christine M. Sorenson, Curtis R. Brandt

The fifth author’s name is incorrect. The correct name is: Nader Sheibani. The correct citation is: Kolb AW, Sauter MM, Ferguson S, Jain AS, Sheibani N, Sorenson CM, et al. (2026) Herpes simplex virus type-1 ocular infection in thrombospondin-1 deficient C57BL/6J mice. PLoS One 21(7): e0352457. https://doi.org/10.1371/journal.pone.0352457.
