# Supplementary material for: Herpes simplex virus type-1 ocular infection in thrombospondin-1 deficient C57BL/6J mice
Source: PLoS One. 2026 Jul 20;21(7):e0352457. doi: 10.1371/journal.pone.0352457 (PMC13489149; doi:10.1371/journal.pone.0352457)
Supplement: Supplementary file 1 [file pone.0352457.s003.pdf]

Reviewer's Comments

Manuscript No.: PONE-D-26-13389
Title: Herpes Simplex Virus Type-1 Ocular Infection in Thrombospondin-1 Deficient Mice.
Authors: Aaron W. Kolb *et al*.

Comments:
The manuscript entitled '*Herpes Simplex Virus Type-1 Ocular Infection in Thrombospondin-1 Deficient Mice'*, aims to determine if loss of Thrombospondin-1 (TSP-1) alters the pathology of HSV-1 keratitis in TSP-1 null (*Thbs1*$^{-/-}$; C57BL/6J background, 8-week-old) mice using a low (INV-2C)- or a high (Ikey)-virulent strains of HSV-1. The outcomes were evaluated in terms of ocular viral titers, severity of blepharitis, corneal neovascularization, corneal clouding, loss of corneal nerves, weight loss, mortality, and quantification of infiltrating cells and T-cell responses in the aqueous and vitreous humors. Based on their observations, authors concluded that lack of TSP-1 did not affect the severity of corneal disease, immune responses to HSV-1, or sensory nerve loss, suggesting either TSP-1 functions are not involved in HSV-1 pathogenesis or that other members of the TSP family can substitute for the loss of TSP-1. While the manuscript is well-written, non-involvement of TSP-1 in HSV-1-induced ocular infection, as concluded in this study, needs more supporting evidences, whereas possible involvement of other members of the TSP family appears inconclusive based on the experiments performed in the present study.

Given the fact that most of the results from this study were against the author's own expectations and pre-established findings, rather these turned out to be a surprise (e.g., as mentioned at lines 422-423) for authors themselves, the roles of suitable controls and due characterization of the animals and reagents used in this study become critical and following clarifications/queries are natural to accept the author's claim. Please consider addressing following comments:

1. Why were the C57BL/6J mice chosen for this study while a previous publication from author's own group (Kolb AW et al., PLoS One, 2023, PMID: 37319284) reports that '*The BALB/c inbred mouse strain is widely used in ocular HSV-1 studies……and is more susceptible to HSV-1 corneal disease than C57BL/6'*? Was the purpose here to check the suitability of C57BL/6J mice for development of HSV-1 infection as a model? If so, the title of the manuscript may, more appropriately, be reframed as '*Herpes Simplex Virus Type-1 Ocular Infection in Thrombospondin-1 Deficient* ***C57BL/6J*** *Mice"*. The title, if required, may further be revised to better represent the results obtained in this study.
2. Was the virulence of the prepared viral stocks and strains confirmed with appropriate controls? If yes, please provide the data as supplementary information.
3. Please refer to the section 'Animals' at lines 133-143 and answer following queries:
   - Line 134: What was the source of TSP-1 null mice used in this study? It is unclear that whether these mice were received from the cited group of Dr. Jack Lawler in Boston, produced in-house, or were procured from commercial sources.
   - Complete characterization of the TSP-1 null mice (used in this study) including the absence of Thrombospondin-1 (*Thbs1)* at both the transcript and protein levels in mice tissues (particularly in the corneas/eye balls) is critical to support the author's claim. Batch-to-batch genetic variations, sometimes acquired during breeding, may affect the copy number and distribution of *Thbs1* gene on alleles. While it is

understood that authors must have verified the genetic background of the THP-1 null mice in their own laboratory setup before performing the experiments, the availability of this data may convince the readers.

- Line 135: What do the Rd1 and Rd8 mutations signify here? Please explain.
- Lines 134-136: Since it is mentioned that 'TSP-1 null mice and control C57BL/6J were **bred** in-house using standard procedures….', were the genotypes of the each mouse/progenies involved **in this study** confirmed as *Thbs1*$^{-/-}$ vs. *Thbs*$^{+/+}$*?* Even, a heterozygote *Thbs*$^{+/-}$ may significantly affect the study outcomes. Please provide the genotyping data as supplementary information. Unfortunately, the link given for data availability at lines 468-469 did not work.
- Lines 137-138: Why was there a random distribution of mice between the two viral strains (15 mice per group for the INV-2C virus vs. 14 C57BL/6J control and 19 *Thbs1*$^{-/-}$ *for Ikey* strain)?

4. Please refer to lines 146-147: '…followed by application of a 5μl drop of DMEM with 2% serum containing 1x10^{6} PFU of virus.' Why was the serum used here? What was its origin: bovine or human? Was it defined or undefined? Please provide these details including manufacturer, catalogue number, and lot number of the serum used in this study as serum may contain TSP-1 and even traces of undefined serum may affect the outcomes of this study. In case, a defined serum was used (as mentioned later at lines 162 under section 'Measurement of Ocular Viral Titers': '..serum (1:1 defined supplemented calf serum/fetal bovine serum)…') please provide the composition. Use of PBS or only DMEM might have been preferred.
5. Please refer to lines 165-166, under section Corneal Nerve Loss: 'The eyes from…………harvested on days 2 and 7 PI and fixed'. Why were these timepoints (day 2 and day 7, Post-infection) chosen? Please provide the rationale.
6. Lines 209-211, Section FACS Analysis: 'Note, the critical comparison is between the infected C57BL/6J mice and infected TSP-1$^{-/-}$ mice and not non-infected vs. infected mice so to reduce the number of animals we did not include uninfected mice'. While this statement appears justified, it would have been better to include uninfected mice too as negative control since the results obtained are against author's own expectations and the established role of TSP-1.
7. Lines 266-269: If possible, please provide the data supporting the statement 'There were some mice that developed stromal keratitis scores………….was not significantly different between the two viruses'
8. Line 273: 'Two viral strains, INV-2C and Ikey…..'. Please use the same standard format 'Ikey' or 'IKEY' throughout the manuscript.
9. Lines 277-278: 'Note that the scoring of eye disease was halted at day 7 PI for Ikey infected eyes because they were sacrificed for histology and immune studies.'. Why were these mice sacrificed at day 7? Please provide the rationale.
10. Lines 370-372/Table 1: Please explain the possible reasons for randomly increased counts in Eye 2 in both the Anterior and Posterior segments which appear significantly higher compared with other eyes in the group.
11. Line 267: 'that developed stromal keratitis………….in both CC57BL/6J control….'. Please remove an additional 'C' before C57BL/6J.

12. Figures: Since both the wild type and TSP-1 null mice were of the C57BL/6J background, it may be appropriate to group the results as 'Wild Type' and 'Thbs1$^{-/-}$null' mice for easier comprehension in all the figures, as applicable.
13. Figure 1: It would be helpful and more convincing to provide the representative images of the eyes that were included in scoring of different ocular disease parameters.
14. Figure 5: Please label the corresponding regions in tissue sections and indicate the areas (in Figs. 5A and 5D) which represent magnified Figs. 5B-C, and 5E-F. If possible, a lowest possible magnification (e.g., 4x) image may be included to appreciate gross histological changes, if any, in the corresponding sections of the murine eyes.
