# Supplementary material for: Herpes simplex virus type-1 ocular infection in thrombospondin-1 deficient C57BL/6J mice
Source: PLoS One. 2026 Jul 20;21(7):e0352457. doi: 10.1371/journal.pone.0352457 (PMC13489149; doi:10.1371/journal.pone.0352457)
Supplement: Supplementary file 2 [file pone.0352457.s005.docx]

Response to Reviewer Comments

PONE-D-13389

Herpes Simplex Virus Type-1 Ocular Infection in Thrombospondin-1-Deficient Mice

Why were C57BL/6 mice chosen? The Thbs-/- mice were generated on the C57/BL6 background. To do these studies in BALB/C mice we would have had to cross the null mice with WT BALB/C mice and then back cross for several generations. This would have taken up to a year at least. We added the mouse strain in the title. Note, the mutant mice do not reproduce well so experiments were done and as cohorts reached 8 weeks old, we used whatever mice were available.

1. Virulence of the viral strains. The virulence properties of INV-2C were published previously (Kolb et. al., 2023). We have included a Supplemental Figure 1 showing the virulence properties of the Ikey strain in BALB/C mice since we have not previously published that data. After infecting the mice, we titered the diluted stock to make sure the inoculum was correct.
2. Source of the mice. The Thbs1-/- mice were obtained commercially and then were bred in house in Dr. Sheibani’s laboratory. He has published several papers using these mice to study retinal vascularization. The mice are routinely genotyped by PCR to confirm the mutation. We have included a Supplementary Figure 2 showing genotyping PCR results and RT-PCR of RNA isolated from the eyes of the Thbs1-/- mice. The data show all KO mice are homozygous for the KO mutation.
3. The strain of C57BL/6 mice originally used to generate several KO strains was found to contain mutations in two genes (Rd1 and Rd8) that cause retinal degeneration. Once that was identified, subsequent KO mice were generated for use in vision research using a C57BL/6 mouse line that were WT for the two mutations. It is now standard in vision research to use KO mice that are WT for the two genes.
4. Why was serum (2%) included in the inoculum. The sera used are all bovine. We have included the supplier, catalog numbers and lots used in the studies in the Methods section. The viral stocks are prepared with 2% serum, and we include it in the final dilution for infection to help stabilize the virus particles. We are using topical application to the cornea and the drop of virus placed is rapidly lost. Eyelid blinking and lachrymal drainage of the solution occurs very rapidly, and the corneas continue to be flushed as the study proceeds so little to no TSP in the serum would persist and affect the results.
5. Why were days 2 and 7 used for nerve analysis? Corneal axon loss is evident within 24 to 48 hours of infection and by day 7 PI most, if not all the axons are lost. These timepoints were a reasonable balance between timepoints to sample and reducing the numbers of animals used.
6. FACS analysis of spleen cells. Numerous papers comparing uninfected spleens to infected spleens have been published by people studying the detailed immunology of HSV infections. We didn’t include this group because the critical comparison is infection of both strains of mice and to reduce the number of animals that were used in the study. As noted above, the KO mice do not reproduce well so the numbers of mice were limited.
7. Some mice developed keratitis. In any animal experiment there is variability in the response and in this case a few of the mice in each group developed mild corneal disease, but the scores and numbers of mice involved were low in both groups and they were not significantly different. This is a minor point, so we removed the comment.
8. Ikey format. The difference was in the labeling of the figure, so we corrected this.
9. Why were Ikey infected mice sacrificed at day 7? At that point the ocular disease scores were very low and were not increasing so we decided to use the eye for the immune studies. This was also done to reduce the number of animals needed. Note for the FACS, antibody titer and ELISPOT studies the mice were harvested at 30 days PI to allow for the generation of an immune response. We did not score the eye disease in these mice.
10. Cell counts. For both strains of mice, we found that 2/3 eyes had much higher numbers of infiltrating cells and 1/3 mice had much less infiltrate. This was not surprising and is the result of normal variability when doing infection studies. We have removed the table and carried out a statistical analysis on the data. This was added to the text of the results section as a statement. There was no significant difference in the mean number of infiltrating cells between the WT and Thbs-/- mice.
11. We corrected the typo (extra C) in front of the mouse designation on line 267.
12. Since we added C57BL/6 to the title we felt it was more appropriate to keep the designation in the figures.
13. We have previously published images of the infected eyes showing the severity of disease and associated scores. Other laboratories have used the same, or very similar scoring scales and also published images of the infected eyes so this is an established and validated system.
14. We added a low power image of the entire eyes to Figure 5 and improved the labeling for various ocular structures.
15. Regarding the Dryad link for the posted data. The link in the manuscript is not active until we notify them the paper has been published so the reviewer was not able to use it. We have subsequently discovered that Dryad provides a special link for this circumstance. It is [http://datadryad.org/share/LINK_NOT_FOR_PUBLICATION/KYj69-hYjdOjQzCEXanySpxsNLhCO4_u0DJDwGy2uZE](https://urldefense.com/v3/__http:/h52wvrds.r.us-west-2.awstrack.me/L0/http:*2F*2Fdatadryad.org*2Fshare*2FLINK_NOT_FOR_PUBLICATION*2FKYj69-hYjdOjQzCEXanySpxsNLhCO4_u0DJDwGy2uZE/1/0101019cd887ccdc-1b5b6eea-b9fd-4bc1-a21d-703f52f8faaa-000000/uuDRTYIO5yxyMJXH1kEdJOV4H6E=469__;JSUlJSU!!Mak6IKo!JcyISHo_3vdqHiTzJQ-sOiCBpHlEyuKWLfY4WV2_HymVFu-PHv-8n96aiOqvq3M9G8Eai8AeDw7SxfXzEe9ETl3rlecM$)
16. The addition of the two supplemental figures and other changes in the manuscript have improved the presentation. Hopefully these changes make the manuscript acceptable for publication.
