# Supplementary material for: Herpes simplex virus type-1 ocular infection in thrombospondin-1 deficient C57BL/6J mice
Source: PLoS One. 2026 Jul 20;21(7):e0352457. doi: 10.1371/journal.pone.0352457 (PMC13489149; doi:10.1371/journal.pone.0352457)
Supplement: Peer review history

# Peer review: Decision Letter 0

**DOI:** 10.1371/journal.pone.0352457.r001

**Authors:** Deepak Shukla

20 Apr 2026

PONE-D-26-13389

Herpes Simplex Virus Type-1 Ocular Infection in Thrombospondin-1 Deficient Mice.

PLOS One

Dear Dr. Brandt,

Thank you for submitting your manuscript to PLOS ONE. After careful consideration, we feel that it has merit but does not fully meet PLOS ONE’s publication criteria as it currently stands. Therefore, we invite you to submit a revised version of the manuscript that addresses the points raised by the reviewers.

Please submit your revised manuscript by Jun 04 2026 11:59PM. If you will need more time than this to complete your revisions, please reply to this message or contact the journal office at plosone@plos.org. When you're ready to submit your revision, log on to https://www.editorialmanager.com/pone/ and select the 'Submissions Needing Revision' folder to locate your manuscript file.

Please include the following items when submitting your revised manuscript:

- A letter that responds to each point raised by the academic editor and reviewer(s). You should upload this letter as a separate file labeled 'Response to Reviewers'.
- A marked-up copy of your manuscript that highlights changes made to the original version. You should upload this as a separate file labeled 'Revised Manuscript with Track Changes'.
- An unmarked version of your revised paper without tracked changes. You should upload this as a separate file labeled 'Manuscript'.

If you would like to make changes to your financial disclosure, please include your updated statement in your cover letter. Guidelines for resubmitting your figure files are available below the reviewer comments at the end of this letter.

If applicable, we recommend that you deposit your laboratory protocols in protocols.io to enhance the reproducibility of your results. Protocols.io assigns your protocol its own identifier (DOI) so that it can be cited independently in the future. For instructions see: https://journals.plos.org/plosone/s/submission-guidelines#loc-laboratory-protocols. Additionally, PLOS ONE offers an option for publishing peer-reviewed Lab Protocol articles, which describe protocols hosted on protocols.io. Read more information on sharing protocols at https://plos.org/protocols?utm_medium=editorial-email&utm_source=authorletters&utm_campaign=protocols.

As the corresponding author, your ORCID iD is verified in the submission system and will appear in the published article. PLOS supports the use of ORCID, and we encourage all coauthors to register for an ORCID iD and use it as well. Please encourage your coauthors to verify their ORCID iD within the submission system before final acceptance, as unverified ORCID iDs will not appear in the published article. *Only*  the individual author can complete the verification step; PLOS staff *cannot*  verify ORCID iDs on behalf of authors.

We look forward to receiving your revised manuscript.

Kind regards,

Deepak Shukla

Academic Editor

PLOS One

Journal Requirements:

When submitting your revision, we need you to address these additional requirements.

1. Please ensure that your manuscript meets PLOS ONE's style requirements, including those for file naming. The PLOS ONE style templates can be found at

https://journals.plos.org/plosone/s/file?id=wjVg/PLOSOne_formatting_sample_main_body.pdf and

https://journals.plos.org/plosone/s/file?id=ba62/PLOSOne_formatting_sample_title_authors_affiliations.pdf

2. To comply with PLOS One submissions requirements, in your Methods section, please provide additional information regarding the experiments involving animals and ensure you have included details on (1) methods of sacrifice, (2) methods of anesthesia and/or analgesia, and (3) efforts to alleviate suffering.

3. Thank you for stating the following in the Acknowledgments Section of your manuscript:

“This work was funded by the Wisconsin Medical Foundation Professorship (CRB), an unrestricted grant to the Department of Ophthalmology and Visual Sciences from Research to Prevent Blindness, Inc., Core grant for Vision Research (P30EY016665), shared instrumentation grants S10OD026957 and S10OD23526, and the TRIP laboratory in the Department of Pathology and Laboratory Medicine (UWCCC-P30CA014520). The work in the NS laboratory is supported by the Retina Research Foundation Professorship. The work in the CMS laboratory is supported by a grant from the Retina Research Foundation.”

We note that you have provided funding information that is not currently declared in your Funding Statement. However, funding information should not appear in the Acknowledgments section or other areas of your manuscript. We will only publish funding information present in the Funding Statement section of the online submission form.

Please remove any funding-related text from the manuscript and let us know how you would like to update your Funding Statement. Currently, your Funding Statement reads as follows:

“Funding: This work was funded by the Wisconsin Medical Foundation Professorship (CRB), an unrestricted grant to the Department of Ophthalmology and Visual Sciences from Research to Prevent Blindness, Inc., Core grant for Vision Research (P30EY016665), shared instrumentation grants S10OD026957 and S10OD23526, and the TRIP laboratory in the Department of Pathology and Laboratory Medicine (UWCCC-P30CA014520). The work in the NS laboratory is supported by the Retina Research Foundation Professorship. The work in the CMS laboratory is supported by a grant from the Retina Research Foundation.”

Please include your amended statements within your cover letter; we will change the online submission form on your behalf.

4. We note that the grant information you provided in the ‘Funding Information’ and ‘Financial Disclosure’ sections do not match.

When you resubmit, please ensure that you provide the correct grant numbers for the awards you received for your study in the ‘Funding Information’ section.

5. Thank you for stating the following financial disclosure:

“Funding: This work was funded by the Wisconsin Medical Foundation Professorship (CRB), an unrestricted grant to the Department of Ophthalmology and Visual Sciences from Research to Prevent Blindness, Inc., Core grant for Vision Research (P30EY016665), shared instrumentation grants S10OD026957 and S10OD23526, and the TRIP laboratory in the Department of Pathology and Laboratory Medicine (UWCCC-P30CA014520). The work in the NS laboratory is supported by the Retina Research Foundation Professorship. The work in the CMS laboratory is supported by a grant from the Retina Research Foundation.”

Please state what role the funders took in the study.  If the funders had no role, please state: "The funders had no role in study design, data collection and analysis, decision to publish, or preparation of the manuscript."

If this statement is not correct you must amend it as needed.

Please include this amended Role of Funder statement in your cover letter; we will change the online submission form on your behalf.

6. Please expand the acronym “NIH” (as indicated in your financial disclosure) so that it states the name of your funders in full.

This information should be included in your cover letter; we will change the online submission form on your behalf.

7. We note that your Data Availability Statement is currently as follows: “All relevant data are within the manuscript and its Supporting Information files”

Please confirm at this time whether or not your submission contains all raw data required to replicate the results of your study. Authors must share the “minimal data set” for their submission. PLOS defines the minimal data set to consist of the data required to replicate all study findings reported in the article, as well as related metadata and methods (https://journals.plos.org/plosone/s/data-availability#loc-minimal-data-set-definition).

For example, authors should submit the following data:

- The values behind the means, standard deviations and other measures reported;

- The values used to build graphs;

- The points extracted from images for analysis.

Authors do not need to submit their entire data set if only a portion of the data was used in the reported study.

If your submission does not contain these data, please either upload them as Supporting Information files or deposit them to a stable, public repository and provide us with the relevant URLs, DOIs, or accession numbers. For a list of recommended repositories, please see https://journals.plos.org/plosone/s/recommended-repositories.

If there are ethical or legal restrictions on sharing a de-identified data set, please explain them in detail (e.g., data contain potentially sensitive information, data are owned by a third-party organization, etc.) and who has imposed them (e.g., an ethics committee). Please also provide contact information for a data access committee, ethics committee, or other institutional body to which data requests may be sent. If data are owned by a third party, please indicate how others may request data access.

8. Please amend the manuscript submission data (via Edit Submission) to include author Christine M. Sorenson.

9. Please amend your authorship list in your manuscript file to include author Christine M. Sorensen.

10. We note that you have referenced “unpublished observations” which has currently not yet been accepted for publication. Please remove this from your References and amend this to state in the body of your manuscript: as detailed online in our guide for authors

http://journals.plos.org/plosone/s/submission-guidelines#loc-reference-style

11. If the reviewer comments include a recommendation to cite specific previously published works, please review and evaluate these publications to determine whether they are relevant and should be cited. There is no requirement to cite these works unless the editor has indicated otherwise.

12. Please review your reference list to ensure that it is complete and correct. If you have cited papers that have been retracted, please include the rationale for doing so in the manuscript text, or remove these references and replace them with relevant current references. Any changes to the reference list should be mentioned in the rebuttal letter that accompanies your revised manuscript. If you need to cite a retracted article, indicate the article’s retracted status in the References list and also include a citation and full reference for the retraction notice.

Additional Editor Comments:

While Reviewer 1 was satisfied with the conclusions drawn, Reviewer 2 has provided a detailed and thoughtful evaluation, and we appreciate the care taken in assessing the manuscript. We agree that several of the points raised will help strengthen the clarity and rigor of the study. The authors are encouraged to address the points where quickly feasible, and where not, acknowledge any limitations or their viewpoints in the Discussion.

[Note: HTML markup is below. Please do not edit.]

Reviewers' comments:

Reviewer's Responses to Questions

**Comments to the Author**

1. Is the manuscript technically sound, and do the data support the conclusions?

The manuscript must describe a technically sound piece of scientific research with data that supports the conclusions. Experiments must have been conducted rigorously, with appropriate controls, replication, and sample sizes. The conclusions must be drawn appropriately based on the data presented.

Reviewer #1: Yes

Reviewer #2: Partly

**********

2. Has the statistical analysis been performed appropriately and rigorously?

Reviewer #1: Yes

Reviewer #2: I Don't Know

**********

3. Have the authors made all data underlying the findings in their manuscript fully available?

The PLOS Data policy requires authors to make all data underlying the findings described in their manuscript fully available without restriction, with rare exception (please refer to the Data Availability Statement in the manuscript PDF file). The data should be provided as part of the manuscript or its supporting information, or deposited to a public repository. For example, in addition to summary statistics, the data points behind means, medians and variance measures should be available. If there are restrictions on publicly sharing data—e.g. participant privacy or use of data from a third party—those must be specified.

Reviewer #1: Yes

Reviewer #2: No

**********

4. Is the manuscript presented in an intelligible fashion and written in standard English?

PLOS ONE does not copyedit accepted manuscripts, so the language in submitted articles must be clear, correct, and unambiguous. Any typographical or grammatical errors should be corrected at revision, so please note any specific errors here.

Reviewer #1: Yes

Reviewer #2: Yes

**********

5. Review Comments to the Author

Please use the space provided to explain your answers to the questions above. You may also include additional comments for the author, including concerns about dual publication, research ethics, or publication ethics. (Please upload your review as an attachment if it exceeds 20,000 characters)

Reviewer #1: This study investigates whether loss of thrombospondin-1 influences the severity of ocular HSV-1 infection. Using both recombinant and clinical HSV-1 strains in Thbs1⁻/⁻ and control mice, the authors found no significant differences in keratitis severity, corneal neovascularization, viral load, immune responses, or corneal nerve loss between groups. These findings suggest that TSP-1 is not essential for HSV-1 corneal pathogenesis.

A minor suggestion would be to perform statistical analysis for data in Figure 3.

Reviewer #2: Please see the review report for detailed comments. Given the fact that most of the results from this study were against the author’s own expectations and pre-established findings, rather these turned out to be a surprise for authors themselves, the roles of suitable controls and due characterization of the animals and reagents used in this study become critical to accept authors claim.

**********

6. PLOS authors have the option to publish the peer review history of their article (what does this mean?). If published, this will include your full peer review and any attached files.

If you choose “no”, your identity will remain anonymous but your review may still be made public.

**Do you want your identity to be public for this peer review?**  For information about this choice, including consent withdrawal, please see our Privacy Policy.

Reviewer #1: No

Reviewer #2: No

**********

[NOTE: If reviewer comments were submitted as an attachment file, they will be attached to this email and accessible via the submission site. Please log into your account, locate the manuscript record, and check for the action link "View Attachments". If this link does not appear, there are no attachment files.]

To ensure your figures meet our technical requirements, please review our figure guidelines: https://journals.plos.org/plosone/s/figures

You may also use PLOS’s free figure tool, NAAS, to help you prepare publication quality figures: https://journals.plos.org/plosone/s/figures#loc-tools-for-figure-preparation.

NAAS will assess whether your figures meet our technical requirements by comparing each figure against our figure specifications.

Attachment Submitted filename: Review Report_PONE-D-26-13389.pdf

# Author comment: Author response to Decision Letter 1

**DOI:** 10.1371/journal.pone.0352457.r002

22 May 2026

Response to Reviewer Comments

PONE-D-13389

Herpes Simplex Virus Type-1 Ocular Infection in Thrombospondin-1-Deficient Mice

1. Why were C57BL/6 mice chosen? The Thbs-/- mice were generated on the C57/BL6 background. To do these studies in BALB/C mice we would have had to cross the null mice with WT BALB/C mice and then back cross for several generations. This would have taken up to a year at least. We added the mouse strain in the title. Note, the mutant mice do not reproduce well so experiments were done and as cohorts reached 8 weeks old, we used whatever mice were available.

2. Virulence of the viral strains. The virulence properties of INV-2C were published previously (Kolb et. al., 2023). We have included a Supplemental Figure 1 showing the virulence properties of the Ikey strain in BALB/C mice since we have not previously published that data. After infecting the mice, we titered the diluted stock to make sure the inoculum was correct.

3. Source of the mice. The Thbs1-/- mice were obtained commercially and then were bred in house in Dr. Sheibani’s laboratory. He has published several papers using these mice to study retinal vascularization. The mice are routinely genotyped by PCR to confirm the mutation. We have included a Supplementary Figure 2 showing genotyping PCR results and RT-PCR of RNA isolated from the eyes of the Thbs1-/- mice. The data show all KO mice are homozygous for the KO mutation.

4. The strain of C57BL/6 mice originally used to generate several KO strains was found to contain mutations in two genes (Rd1 and Rd8) that cause retinal degeneration. Once that was identified, subsequent KO mice were generated for use in vision research using a C57BL/6 mouse line that were WT for the two mutations. It is now standard in vision research to use KO mice that are WT for the two genes.

5. Why was serum (2%) included in the inoculum. The sera used are all bovine. We have included the supplier, catalog numbers and lots used in the studies in the Methods section. The viral stocks are prepared with 2% serum, and we include it in the final dilution for infection to help stabilize the virus particles. We are using topical application to the cornea and the drop of virus placed is rapidly lost. Eyelid blinking and lachrymal drainage of the solution occurs very rapidly, and the corneas continue to be flushed as the study proceeds so little to no TSP in the serum would persist and affect the results.

6. Why were days 2 and 7 used for nerve analysis? Corneal axon loss is evident within 24 to 48 hours of infection and by day 7 PI most, if not all the axons are lost. These timepoints were a reasonable balance between timepoints to sample and reducing the numbers of animals used.

7. FACS analysis of spleen cells. Numerous papers comparing uninfected spleens to infected spleens have been published by people studying the detailed immunology of HSV infections. We didn’t include this group because the critical comparison is infection of both strains of mice and to reduce the number of animals that were used in the study. As noted above, the KO mice do not reproduce well so the numbers of mice were limited.

8. Some mice developed keratitis. In any animal experiment there is variability in the response and in this case a few of the mice in each group developed mild corneal disease, but the scores and numbers of mice involved were low in both groups and they were not significantly different. This is a minor point, so we removed the comment.

9. Ikey format. The difference was in the labeling of the figure, so we corrected this.

10. Why were Ikey infected mice sacrificed at day 7? At that point the ocular disease scores were very low and were not increasing so we decided to use the eye for the immune studies. This was also done to reduce the number of animals needed. Note for the FACS, antibody titer and ELISPOT studies the mice were harvested at 30 days PI to allow for the generation of an immune response. We did not score the eye disease in these mice.

11. Cell counts. For both strains of mice, we found that 2/3 eyes had much higher numbers of infiltrating cells and 1/3 mice had much less infiltrate. This was not surprising and is the result of normal variability when doing infection studies. We have removed the table and carried out a statistical analysis on the data. This was added to the text of the results section as a statement. There was no significant difference in the mean number of infiltrating cells between the WT and Thbs-/- mice.

12. We corrected the typo (extra C) in front of the mouse designation on line 267.

13. Since we added C57BL/6 to the title we felt it was more appropriate to keep the designation in the figures.

14. We have previously published images of the infected eyes showing the severity of disease and associated scores. Other laboratories have used the same, or very similar scoring scales and also published images of the infected eyes so this is an established and validated system.

15. We added a low power image of the entire eyes to Figure 5 and improved the labeling for various ocular structures.

16. Regarding the Dryad link for the posted data. The link in the manuscript is not active until we notify them the paper has been published so the reviewer was not able to use it. We have subsequently discovered that Dryad provides a special link for this circumstance. It is http://datadryad.org/share/LINK_NOT_FOR_PUBLICATION/KYj69-hYjdOjQzCEXanySpxsNLhCO4_u0DJDwGy2uZE

17. The addition of the two supplemental figures and other changes in the manuscript have improved the presentation. Hopefully these changes make the manuscript acceptable for publication.

Attachment Submitted filename: Response to Reviewer Comments THP-1.docx

# Editor report: Decision Letter 1

**DOI:** 10.1371/journal.pone.0352457.r003

**Authors:** Deepak Shukla

10 Jun 2026

Herpes Simplex Virus Type-1 Ocular Infection in Thrombospondin-1 Deficient C57BL/6J Mice.

PONE-D-26-13389R1

Dear Dr. Brandt,

We’re pleased to inform you that your manuscript has been judged scientifically suitable for publication and will be formally accepted for publication once it meets all outstanding technical requirements.

Within one week, you’ll receive an e-mail detailing the required amendments. When these have been addressed, you’ll receive a formal acceptance letter and your manuscript will be scheduled for publication.

An invoice will be generated when your article is formally accepted. Please note, if your institution has a publishing partnership with PLOS and your article meets the relevant criteria, all or part of your publication costs will be covered. Please make sure your user information is up-to-date by logging into Editorial Manager at Editorial Manager® and clicking the ‘Update My Information' link at the top of the page. For questions related to billing, please contact billing support.

If your institution or institutions have a press office, please notify them about your upcoming paper to help maximize its impact. If they’ll be preparing press materials, please inform our press team as soon as possible -- no later than 48 hours after receiving the formal acceptance. Your manuscript will remain under strict press embargo until 2 pm Eastern Time on the date of publication. For more information, please contact onepress@plos.org.

Kind regards,

Deepak Shukla

Academic Editor

PLOS One

Additional Editor Comments (optional):

Reviewers' comments:

# Editor report: Acceptance letter

**DOI:** 10.1371/journal.pone.0352457.r004

**Authors:** Deepak Shukla

PONE-D-26-13389R1

PLOS One

Dear Dr. Brandt,

I'm pleased to inform you that your manuscript has been deemed suitable for publication in PLOS One. Congratulations! Your manuscript is now being handed over to our production team.

At this stage, our production department will prepare your paper for publication. This includes ensuring the following:

* All references, tables, and figures are properly cited

* All relevant supporting information is included in the manuscript submission,

* There are no issues that prevent the paper from being properly typeset

You will receive further instructions from the production team, including instructions on how to review your proof when it is ready. Please keep in mind that we are working through a large volume of accepted articles, so please give us a few days to review your paper and let you know the next and final steps.

Lastly, if your institution or institutions have a press office, please let them know about your upcoming paper now to help maximize its impact. If they'll be preparing press materials, please inform our press team within the next 48 hours. Your manuscript will remain under strict press embargo until 2 pm Eastern Time on the date of publication. For more information, please contact onepress@plos.org.

You will receive an invoice from PLOS for your publication fee after your manuscript has reached the completed accept phase. If you receive an email requesting payment before acceptance or for any other service, this may be a phishing scheme. Learn how to identify phishing emails and protect your accounts at https://explore.plos.org/phishing.

If we can help with anything else, please email us at customercare@plos.org.

Thank you for submitting your work to PLOS ONE and supporting open access.

Kind regards,

PLOS ONE Editorial Office Staff

on behalf of

Professor Deepak Shukla

Academic Editor

PLOS One
